# Valorization of Vegetable Waste from Leek, Lettuce, and Artichoke to Produce Highly Concentrated Lignocellulose Micro- and Nanofibril Suspensions

**DOI:** 10.3390/nano12244499

**Published:** 2022-12-19

**Authors:** Jose Luis Sanchez-Salvador, Mariana P. Marques, Margarida S. C. A. Brito, Carlos Negro, Maria Concepcion Monte, Yaidelin A. Manrique, Ricardo J. Santos, Angeles Blanco

**Affiliations:** 1 Department of Chemical Engineering and Materials, University Complutense of Madrid, Avda. Complutense s/n, 28040 Madrid, Spain; 2 Laboratory of Separation and Reaction Engineering–Laboratory of Catalysis and Materials (LSRE-LCM), Faculty of Engineering, University of Porto, Rua Dr. Roberto Frias, 4200-465 Porto, Portugal; 3 ALiCE-Associate Laboratory in Chemical Engineering, Faculty of Engineering, University of Porto, Rua Dr. Roberto Frias, 4200-465 Porto, Portugal; 4 Laboratory for Process Engineering, Environment, Biotechnology and Energy (LEPABE), Faculty of Engineering, University of Porto, Rua Dr. Roberto Frias, 4200-465 Porto, Portugal

**Keywords:** lignocellulose microfibrils, cellulose nanofibers, waste recovery, vegetable residues, valorization of agro-wastes

## Abstract

Vegetable supply in the world is more than double than vegetable intake, which supposes a significant waste of vegetables, in addition to the agricultural residues produced. As sensitive food products, the reasons for this waste vary from the use of only a part of the vegetable due to its different properties to the product appearance and market image. An alternative high-added-value application for these wastes rich in cellulose could be the reduction in size to produce lignocellulose micro- and nanofibrils (LCMNF). In this sense, a direct treatment of greengrocery waste (leek, lettuce, and artichoke) to produce LCMNFs without the extraction of cellulose has been studied, obtaining highly concentrated suspensions, without using chemicals. After drying the wastes, these suspensions were produced by milling and blending at high shear followed by several passes in the high-pressure homogenizer (up to six passes). The presence of more extractives and shorter fiber lengths allowed the obtention of 5–5.5% leek LCMNF suspensions and 3.5–4% lettuce LCMNF suspensions, whereas for artichoke, only suspensions of under 1% were obtained. The main novelty of the work was the obtention of a high concentration of micro- and nanofiber suspension from the total waste without any pretreatment. These high concentrations are not obtained from other raw materials (wood or annual plants) due to the clogging of the homogenizer, requiring the dilution of the sample up to 1% or the use of chemical pretreatments.

## 1. Introduction

According to World Health Organization and Food and Agriculture Organization guidelines, the recommended consumption of fruits and vegetables is at least 400 g/day, excluding potatoes and legumes [1]. The majority of countries suggest that at least three of five servings (240 g/day) should come from vegetables [2]. According to Kalmpourtzidou et al. (2020), in 88% of the 162 countries analyzed, vegetable intake was below the recommendations, with a weighted mean of 186 g/day and wide variations between countries from 56 to 349 g/day for different reasons [3]. However, the vegetable supply per country presents a weighted mean of more than double with respect to vegetable intake, with a mean of 431 g/day (71–882 g/day), which is a significant waste, in addition to the agricultural residues produced [3]. This waste is mainly due to vegetables being considered sensitive food products and due to their that their loss can easily occur due to poor practices, degradation, the use of only a part of the vegetable for its different properties, or a poor external appearance [4]. These residues can be produced both in transportation, stores, and homes, and they are an abundant source of cellulose and other compounds, which allows their valorization. The most widespread application examples include biogas production, burning, or extracting valuable compounds. Less common are the production of metallic nanoparticles, biochar, edible films, or the preparation of microbiological media [5,6,7].

On the other hand, cellulose micro- and nanofibrils (CMNFs) have been widely developed in the last decade due to their high number of potential applications [8]. They are usually produced from different raw materials such as wood, non-woody plants, agroforestry residues, or bacteria with multiple process options [9]. Therefore, a possible alternative application of vegetable wastes rich in cellulose, hemicellulose, and lignin could be the reduction in size to produce lignocellulose micro- and nanofibrils (LCMNFs). In recent years, LCMNFs have emerged as a considerable strategy to improve the efficiency of lignocellulose resources, increasing the production yield and also contributing to a higher hydrophobicity regarding CMNFs [10]. Furthermore, the lignin in the cell wall can control the content of water by shielding the free accessible hydroxyl group from forming hydrogen bonds with water molecules, reducing the water retention value [11].

Currently, a fit-for-use approach is being developed to produce CMNFs and LCMNFs, varying the treatments and raw materials according to the most important characteristics that should be prioritized, such as the rheology of the hydrogel formed, the cellulose concentration, the aspect ratio, or production without chemical reagents [12]. Therefore, the presence of other compounds such as lignin, pectin, or extractives could be used as a tool to tailor the properties of CMNFs rather than posing a problem [13].

Some examples of applications in which cellulose nanofibers and these compounds can be used are the case of pectin in 3D bioprinting [14] or the formation of multifunctional structures [15]. Concerning lignin, it is possible to obtain LCMNFs as a reinforcement agent in different polymer matrices as the main application [16,17]. In this case, when the LCMNFs are produced from agroforestry residues, the proportion of lignin is lower than in woody products, favoring the production of LCMNF suspensions. However, agroforestry residues have lower concentrations of pectin than fruit and vegetable waste [7,9]. As for extractives, they are mostly removed in bleached pulps from woody and non-woody raw materials [18], whereas in other cases, extractives are directly extracted from the raw materials without assessing their effect [19,20]. In other cases, specific extractives were added to cellulose nanofibrils (CNFs) to improve their antibacterial and other properties [21,22].

According to the state-of-the-art study, LCMNFs have barely been produced directly from fruit or vegetable greengrocery waste with higher contents of pectin and extractives than in wood pulp, despite the wide range of applications for which LCMNFs can be used [23].

Pectin is a negatively charged heteropolysaccharide that contains different structural elements, mainly homogalacturonan and rhamnogalacturonan, but also other compounds to a very minor extent, like xylose, arabinose, and galactose [7,24]. These polysaccharides have significant content of carboxyl and acetyl groups in their structures that could favor the repulsion between pectin molecules [25]. In some research, raw materials such as sugar beet pulp have been pretreated to remove the pectin almost completely [26,27]. Nevertheless, in a recent article, Hiasa et al. (2016) found that the pectin contained in the raw material (mandarin peel) prevented the aggregation of CMNFs due to the strong interaction between pectin and cellulose. In contrast, the addition of pectin to the CMNF suspension did not prevent the aggregation [23].

Extractives are a multitude of compounds of diverse natures which are soluble in organic solvents or water [28]. The presence of these compounds varies greatly depending on the vegetable selected. For instance, romaine lettuce is rich in organic acids such as quinic or α-keto acids, phenolic acids such as p-coumaric and caffeic acid, folates, carotenoids, and chlorophylls [29]; the leek is rich in pentanol, methyl furan, flavonoids, polysaccharides, glucosinolates, or organosulfur compounds [30,31], whereas artichoke, with a lower extractive content, is rich in phenolic compounds such as cynarin or luteolin, phenolic acids such as caffeic, coumaric, or quinic acid, acid alcohols, or flavonoid glycosides [32].

Thus, it could be thought that the presence of pectin in vegetable waste, which avoids the aggregation of LCMNFs, and extractives, such as phenolic acids incorporated into the cell wall, which can repel each other like the carboxyl groups incorporated to produce CMNFs by carboxylation, both could favor the homogenization of the samples [33]. In this way, a direct homogenization of the waste, avoiding clogging in the equipment or the aggregation of fibers, would facilitate the production of LCMNFs at high concentration without needing chemical pretreatments. This kind of suspension at high concentration cannot be obtained from other raw materials due to the clogging during mechanical treatments, requiring the dilution of the sample, or the use of intensive pretreatments such as the oxidation of the fibers using NaClO as oxidant and using NaBr and TEMPO as catalysts [34,35]. In addition, the increase in the concentration of solids means that during the energetically intensive mechanical treatments, the energy consumed per amount of dry solid is remarkably reduced at the same time as the working time.

Therefore, the aim of this research is the production of highly concentrated LCMNFs by different pretreatments and several homogenization steps, using three types of residues from leek, romaine lettuce, and artichoke, with different pectin and extractive contents in each of them. The novelty stems from the fact that normally a pretreatment is applied to the waste to extract the desirable compounds, mainly the cellulosic fraction, which produces low yields. However, in this paper, we show that a very high-yield process can be successfully applied using only a low mechanical pretreatment with a mill and a blender. Furthermore, the use of only mechanical processes has been studied to avoid chemical reagents, as required in applications such as those related to the food industry, as a stabilizer or in the preparation of emulsions. The properties of these LCMNFs have been characterized and compared with CMNFs from bleached eucalyptus pulp with the same treatments, but with lower contents of extractives and pectin.

## 2. Materials and Methods

**Figure 1:**
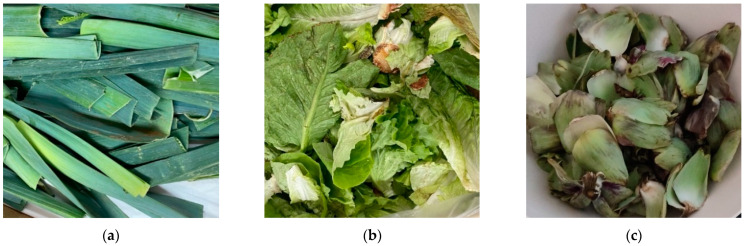
Residues of vegetables: (**a**) leek, (**b**) romaine lettuce, and (**c**) artichoke.

Residues of leek, romaine lettuce, and artichoke were kindly supplied by a local greengrocery in Madrid (Figure 1). Leek residues are the remains of the upper part of the leek stem, which are discarded to reduce the size of each piece. They are highly fibrous and therefore not normally edible. The residues of romaine lettuce are its outermost layers that have a worse appearance and are therefore discarded for sale. Finally, artichoke residues are the outermost layers which are highly fibrous and are usually discarded for consumption. The different residues were only washed with tap water to remove sand and other impurities present in the residues.

To compare the effect of each residue, bleached kraft eucalyptus pulp (Torraspapel S.A., Zaragoza, Spain) with a deficient proportion of extractives and lignin were used as a cellulose source commonly used to produce LCMNFs. Other reagents used for characterization were NaOH, HCl, CaCO_3_, NaCl, cupriethylenediamine, crystal violet, or H_2_SO_4_ supplied by Merck and Poly-l-Lysine solution, obtained from Electron Microscopy Sciences.

The chemical composition of the greengrocery residues (cellulose, pectin, acid soluble and insoluble lignin, hemicellulose, ashes, and extractives) was characterized. Extractive contents were quantified from Soxhlet extraction according to TAPPI T204 [36]. Ash content was determined by calcination at 525 °C according to TAPPI T211 [37]. The other compounds were characterized following NREL/TP-510-42618 standard [38]. The cellulose sample (300 mg) was hydrolyzed with 3 mL of 72 wt. % H_2_SO_4_ for 1 h at 30 °C in a water bath. Then, 84 g of deionized water was added to the sample and introduced in an autoclave for 1 h at 121 °C. Hydrolyzed samples were vacuum filtered and the Klason lignin was determined from the sediment in the filter. On the other hand, the soluble lignin was obtained by measuring the absorbance of the filtrate in the UV-Visible spectrophotometer at 240 nm. Hemicellulose, cellulose, and pectin were analyzed by HPLC from the filtrate after neutralization with CaCO_3_ and filtered through 0.2 μm filters. Finally, the dry content of the residues was determined at 80 °C until constant weight, and carboxyl groups were determined by conductimetric titration using constant doses of 200 µL of 0.05 M NaOH [39].

To produce LCMNFs, five combinations of mechanical pretreatments were studied before the homogenization stages:

- Blender (B): The raw materials were directly crushed with tap water in a blender for 3 min with a total solid content of 7–8%.
- Blender + Dried (BD): Raw materials were crushed under the same conditions as B with a subsequent stage of drying of the material at 105 °C until constant weight.
- Blender + Dried + Milled (BDM): After BD treatment, the residues were ground in a CT 293 Cyclotec mill (Foss, Hilleroed, Denmark) by high-speed action, rolling the sample against the inner circumference of a durable grinding surface and then passing it through a sieve of diameter 1.7 mm.
- Dried + Milled (DM): Raw materials were first dried at 80 °C and then milled in the same laboratory mill as before.
- Dried + Milled + Blender (DMB): The same conditions as DM, with an additional stage mixing the powder with water using the blender for 3 min with a total solid content of 7–8%.

After these pretreatments, a sample of each material produced from the different raw materials was taken and visualized in a Zeiss Axio Lab A1 optical microscope with a camera AxioCam ERc 5s under 5× magnification (Carl Zeiss Microscopy GmbH, Göttingen, Germany) to evaluate the morphology of the fibers. Images were treated to adapt color, brightness, and contrast and also to include the scale bar.

Then, the samples with a small size for each raw material were selected for the main mechanical treatment of high-pressure homogenization (HPH) in the Panda Plus 2000 homogenizer (Gea Niro Soavy, Parma, Italy), carried out at 600 bar and different passes of homogenization. The first HPH stage was prepared in duplicate at the maximum possible concentration of solids. For this purpose, different decreasing concentrations were tested until selecting a concentration of microfibrils that avoided clogging in the homogenizer and with a smooth flow through the equipment.

LCMNFs were characterized by different techniques. First, solid content in the LCMNF suspensions were determined after drying at 80 °C until constant weight. Transmittance readings of LCMNFs diluted at 0.1 wt. % were measured at 600 nm on an LLG-uniSPEC 4 Spectrophotometer (LLG-Labware, Meckenheim, Germany) using distilled water as reference. In order to avoid sedimentation, the suspensions were stirred and immediately analyzed in the spectrophotometer.

Aspect ratio (AR) was obtained by the simplified gel point methodology based on the sedimentation of the fibers at low consistency, as shown in Equation 1 [40]. To prepare the samples, LCMNFs were diluted using deionized water until 250 mL and slowly agitated with a magnetic stirrer for 10 min. Then, a volume of 200 μL of crystal violet 0.1 wt % was added during the agitation to favor the sediment visualization [41]. The suspensions were left to settle into graduated cylinders until they reached a steady value indicating complete deposition of fibers. If the final relative sedimentation height is not in the range of 4–12%, a new sedimentation experiment is required to obtain a better approximation of the gel point [40]. AR was calculated by Equation 2 from the gel point value according to Varanasi et al. (2013) [42], assuming a density of fibers around 1500 kg/m^3^ and using the crowding number theory [43].

(1) $$Gel\;point\;\left(\mathrm{kg}/m^{3}\right)\approx \;\frac{Initial\;suspension\;concentration\;\left(\mathrm{kg}/m^{3}\right)}{Relative\;sedimentation\;height\;}$$

(2) $$Aspect\;Ratio=5.9\cdot \sqrt{\frac{1000}{Gel\;point\;\left(\mathrm{kg}/m^{3}\right)\;}}$$

Polymerization degree was calculated from the limiting viscosity number of the LCMNF suspensions, using cupriethylendiamine as a solvent and determined by the international standard ISO5351/1, based on the Mark–Houwink–Sakurada (MHS) equation [44,45].

Superficial cationic demand was determined by colloidal titration of the diluted LCMNFs at 0.1 wt. %, with 0.001 N polydiallyldimethylammonium chloride, using a Mütek PCD05 particle charge detector (BTG Instruments GmbH, Herrsching, Germany).

Finally, optical micrographs (OMs) of each LCMNF suspension were taken under 5× magnification with the same procedure as the initial suspensions prior to the homogenization. At least 10 micrographs of each LCMNF were taken to study the diameter range of the LCMNFs. The diameter range was measured in ImageJ from the determination of the standard deviation of the diameters and the percentile 10, 50, and 90 using at least 100 samples.

Furthermore, OMs were used to determine the fibrillation degree of the LCMNF suspensions by calculating the branching index (BI) [12,18]. The procedure to calculate the BI includes first the binarization of the images using ImageJ. The fibers and networks were eroded and analyzed in terms of size and shape with the same program according to the procedures described in previous publications [18,46]. The number of nodes and branches in each fiber or network of fibers was determined for each skeletonized image and the projected area of each one in the binarized images. Finally, the slope obtained from the linear regression of the map that relates the number of nodes vs. the projected area was used to measure the BI of each sample.

## 3. Results and Discussion

### 3.1. Chemical Composition of the Raw Materials

The raw materials used to produce LCMNFs are characterized in Table 1. First, raw materials were dried to obtain the total solid content except in eucalyptus, whose pulp was dried in the form of plates. Whereas lettuce is rich in water (94.7%), leek and artichoke waste is more fibrous, with a higher solid content of 11.2 and 28.7%, respectively. As for carboxyl groups, bleached eucalyptus pulps have a very low content of carboxyl groups and extractives. In the case of the waste, the carboxyl groups come mainly from the different extractives of the sample. We highlight their content in lettuce, with a value of 2.51 mmol/g, which also shows the higher content of extractives among the samples analyzed. In this sense, the content of extractives is much higher in lettuce (14.4%) and leek (9.2%) than in eucalyptus or artichokes samples, which contain less than 2% of extractives. The organic soluble extractives present in the fibrils contribute with different carbonyl groups in their structure that may favor the repulsion of the fibrils. The repulsion favors separation when mechanical treatments are applied, in the same way that cellulose from woody plants is oxidized by TEMPO-mediated oxidation to favor the fibers’ repulsion and thus the production of the CNFs [33].

As for the chemical composition of the residues on a dry basis, the high content of ash in the lettuce stands out (27.1%), as reported in literature and in part associated with the fertilizers used, which mostly remain on the external part of the lettuce, which was the part used in this study because it degrades more easily [47]. Ash content of leek is relatively lower (10.8%) and much lower in artichoke (8.2%), with similar results to the literature [32], whereas in the case of eucalyptus it is under 1%. The amount of cellulose is very disparate between the different sources. Eucalyptus pulp treated to maximize cellulose content has 66.5% of cellulose, while leek and artichoke have a cellulose content of around 30%. In contrast, lettuce has a much lower content of cellulose (17.7%), with higher contents of ash and soluble lignin than cellulose. As for the pectin content, galacturonic acid proceeds from pectin, but pectin is also part of rhamnose, which also comes from hemicelluloses to a lesser extent [48,49]. Regarding the content of pectin (galacturonic acid and rhamnose), it can be observed that the three waste products have a pectin content higher than 10%, reaching 17% in the artichoke. In contrast, the reference eucalyptus pulp has a pectin content under 8%. Hemicelluloses (formed in the chemical composition by arabinose, the overlapping of xylose, mannose, and galactose, and part of the rhamnose content) are part of the support and structural substance of cell walls. The higher content is observed in eucalyptus pulp and artichoke, with 14% on a dry basis, but with different proportions of the polysaccharides. While eucalyptus has a majority proportion of xylose among the hemicelluloses [50], the hemicellulose in artichoke is more heterogeneous, highlighting the arabinose and galactose [51]. Hemicellulose content in leek is almost similar, with around 13% in total, whereas in the case of lettuce, the amount of hemicellulose is ~7%. Finally, total lignin is similar in the three residues, around 25–30%, whereas in the eucalyptus pulp, the residual lignin in the pulp is under 10%, although slightly higher than other studies with bleached eucalyptus pulp [12,52]. The presence of a higher content of lignin to prepare cellulose pulps decreases their production costs and produces a higher thermal stability and a higher chemical versatility, making for higher compatibility with other materials [53]. The ratio between soluble and insoluble lignin in artichoke is equal to the unit, while in the case of lettuce and leek, this ratio is approximately 4:1.

### 3.2. Comparison of Different Pretreatments to Obtain LCMNFs

**Figure 2:**
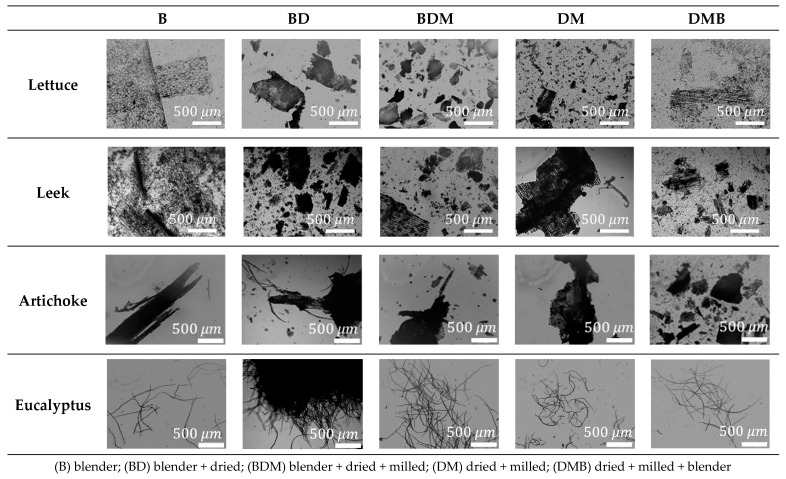
OM images (5× magnification) of cellulose mechanically pretreated.

To obtain highly concentrated LCMNF suspensions, different combinations of pretreatments were evaluated, also considering the shelf life of the suspension after the pretreatment. Five combinations of pretreatments were tested in all raw materials, and OM images were taken and shown in Figure 2.

In the case of lettuce and leek, the use of a high-speed blender (B) produces a good disintegration of both residues with a narrow size distribution, which could be helpful during the HPH process. As for artichoke, this pretreatment produces a more heterogeneous result. Nevertheless, this wet process noticeably reduces the storage life of the waste, requiring a quick production of the LCMNFs to avoid higher degradation. A possible solution could be the subsequent drying (BD) or the drying and milling of the blender samples (BDM), allowing the conservation of the samples for longer periods before homogenization. However, BD and BDM pretreatment sequences produce larger and more compact pieces than blender-only samples, as Figure 2 shows, requiring a new wetting process to avoid clogging the HPH, which is an additional blender stage. Therefore, we first propose the drying and milling of the waste (DM), which is helpful to increase the shelf time of the waste, separating the obtention of the wastes from the production of LCMNFs, which would deteriorate faster if they were not dried. In addition, this would allow the use of seasonal waste throughout the year to produce LCMNFs. Then, for LCMNF production, a soaking stage is carried out in the blender (DMB) that further reduces the size of the fibers and aggregates. Comparing the three wastes with DMB, it is possible to observe a separation of the cellulose fibers in smaller fibers, equal to or even smaller in size than using only B, whereas the eucalyptus pulp with the previous bleaching causes only a slight shortening of the fibers.

With all of that, the two ways to obtain LCMNFs with the best results in size were B and DMB, each one with pros and cons. The use of B pretreatment does not separate the obtention of the waste and the preparation of the LCMNFs due to the degradation, while in the DMB sequence, this is possible but requires a higher number of pretreatments, increasing the production costs.

**Figure 3:**
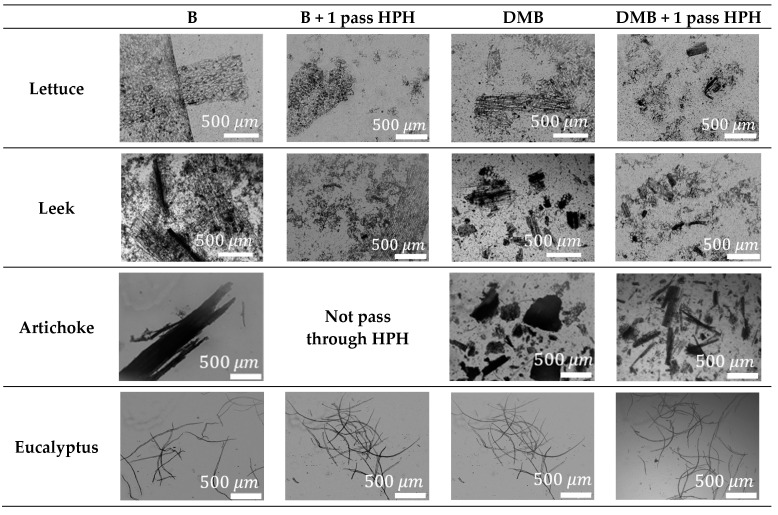
OM images before and after 1 pass of HPH for waste pretreated with the blender (B) or dried, milled, and blender (DMB).

However, the most important parameter to evaluate the effectivity of the pretreatment sequence is the obtention of LCMNF suspensions with the maximum concentration after passing through the HPH. Therefore, the OM images in Figure 3 show the difference between B and DMB before and after one homogenization pass. Table 2 shows the properties of the LCMNFs for the different raw materials.

Lettuce and leek micrographs show a good effectivity of the HPH both in B and DMB. However, the maximum concentration after one pass of HPH is very noticeable between pretreatments. DMB allows a maximum concentration after one pass of HPH of 3.5–4% for lettuce and 5–5.5% for leek, whereas using B, the maximum concentration is under 2%, so the DMB pretreatment is the most effective for the stated purpose. This supposes a large jump in concentration compared to the usual LCMNF suspensions with concentrations under 2% [54]. One exception to this would be the high-purity microcrystalline cellulose from cotton linters in powder [55]. The very low diameter and low aspect ratio of the raw material produces a concentration after 10 passes of HPH of 3.7%, despite the low curl of the initial fibers. On the other hand, in the case of artichoke with larger fibers in the raw material, they do not pass through the HPH at concentrations under 0.2% using B as pretreatment, whereas in the case of DMB, homogenization is possible at 0.9–1%. Finally, the reference samples of eucalyptus pretreated with B and DMB show a maximum consistency after HPH around 0.4–0.5%, more similar to other CNF suspensions treated only mechanically. One of the reasons for this low concentration to pass by the homogenizer is the higher aspect ratio of the eucalyptus fibers, which tends to clog the equipment. Comparing the composition of the four raw materials, those that allowed a higher concentration of solids to produce LCMNFs have a higher content of extractives in the composition, which favors the reduction in dimensions by mechanical treatments.

As for the other characteristics of the suspensions, transmittance was measured at 0.1 wt. % before and after one pass of HPH. The very heterogeneous suspensions of the samples only pretreated produce an easy separation between the solid part and the liquid, with the sedimentation of the solids. This separation is even greater using B as pretreatment, which produces a higher value in transmittance before HPH. This is contrary to what is usual in the production of CNFs, where the transmittance increases as the CNFs fibrillate, starting from micro-sized samples [56]. In all cases, the transmittance is higher using only the blender due to the existence of a more liquid fraction that allows lighter parts to pass through the cell. Furthermore, there are lower transmittance differences with the homogenization samples in the case of DMB because the pretreatment has further destructured the fibers. On the other hand, the aspect ratio is lower using DMB due to the sample passing through the mill, reducing the length of the fibers. Nevertheless, the HPH counterbalances the reduction in the aspect ratio in the laboratory mill. Comparing the different sources, eucalyptus pulp with a higher content of cellulose has the highest aspect ratio, whereas wastes with a more varied composition and heterogeneous pieces have a lower aspect ratio in all cases.

### 3.3. Comparison of LCMNFs with Different Homogenization Passes

Finally, LCMNFs produced with DMB, the best pretreatment selected, and different homogenization passes are evaluated in Table 3. The concentration of solids after the first pass through the homogenizer coincides with the range indicated in Table 2. After more homogenization passes, the amount of solids is maintained, with slight variations depending on whether or not some water evaporates in the homogenizer or if more water has been added between homogenization passes. Table 3 also shows that there is a decrease in transmittance, with the initial homogenization passes showing poor homogeneity of the samples. In the case of lettuce and leek from the third homogenization pass, transmittance increases as expected as the samples become more homogenized [12,55]. In the case of artichoke and eucalyptus, transmittance decreases throughout all passes studied, indicating that the sample is still very heterogeneous.

Polymerization degree, characterized by the measurement of the intrinsic viscosity, was also evaluated. The average number of monomers is much lower in lettuce and leek than in artichoke and eucalyptus. In this sense, the lower degree of polymerization would justify its ease of passing through the homogenizer without clogging. For lettuce, artichoke, and eucalyptus, we observe major differences between the suspension not homogenized and homogenized with one pass, producing the defibrillation and the break of the fibers in the HPH. However, scarce differences were observed from the first pass to the ninth pass for this property. Regarding the case of leek, the polymerization degree is constant for all passes. This relates to what is shown in the leek micrographs in Table 4. As the homogenization passes increase, we observe the separation of leek fibers that are stuck to a greater extent than their shortening. In the case of lettuce and artichoke’s micrographs in Table 4, at low homogenization passes are observed both the shortening and separation of the fibers, whereas at a higher number of passes, the peeling of the fibers and their separation are the predominant effects. With respect to the eucalyptus, we observe a slight shortening and branching of the fibers as the homogenization becomes more severe.

The aspect ratio of LCMNFs increases in all cases with the HPH intensity due to the peeling of the fibers and despite the initial shortening of the fibers. Eucalyptus LCMNFs have the highest aspect ratio due to the sample mainly containing cellulose fibers. Thus, the sample has similar diameter averages to leek or lettuce. Nevertheless, the other wastes with more aggregates from other compounds, such as lignin, have a lower average aspect ratio. As occurs in the polymerization degree, the higher changes are produced in the first pass in the HPH for all cases, whereas in the case of lettuce and leek, the aspect ratio is very similar from three to nine passes. Finally, superficial cationic demand indicates how anionic the LCMNF surface is, and since the treatment is only mechanical, it gives an idea of the increase in surface area of the LCMNFs. As for lettuce and leek, it is possible to observe that the cationic demand value after nine passes of HPH doubled compared to the one in the non-homogenized sample, whereas in artichoke and eucalyptus, the increase is around 50%.

Table 4 also characterizes the diameter range of the fibers shown in the OM at 5× magnification. The resolution of the OM does not allow the visualization of diameters under 3 μm. Nevertheless, images taken in TEM (with diameters under 2 μm) only show a low number of nanofibers, not very representative of the total fibers, with diameters up to 20 nm in leek and lettuce after six and nine homogenization passes. In this regard, the samples are mostly lignocellulose microfibrils. Nevertheless, the diameter range is a valuable tool to evaluate the homogeneity of the sample and the reduction in the size of the larger fibers.

The three residues studied show a higher heterogeneity of the samples than eucalyptus due to the different shapes that they conform to, as the standard deviation shows. As for the longer fibers, they are noticeably reduced with the homogenization steps. In the three wastes studied, after six passes of HPH, the diameter range is almost maintained. Therefore, nine HPH passes are unnecessary in the three cases, as shown from the degree of polymerization or the aspect ratio. In addition, we can observe that despite the smaller diameter of the eucalyptus fibers, the key parameter to avoid clogging in the homogenizer is that the small size goes hand in hand with a short fiber length, as in lettuce and leek.

**Figure 4:**
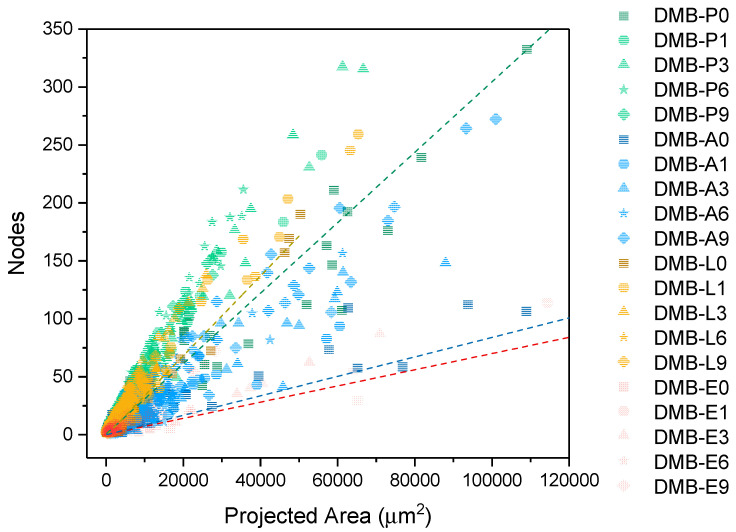
Representation of the map that relates the number of nodes in the eroded images vs. the projected area in the binary images of LCMNF suspensions produced with dried, milled, and blender (DMB) and different homogenization passes.

As for the fibrillation degree of the samples, the representation of the number of nodes in the eroded micrographs vs. the projected area in the binary micrographs has been studied in the map plotted in Figure 4. This map not only gives an idea of fibrillation, but also of the shortening of the fibers and the entanglement [18]. A reduction in the projected area can be observed with the homogenization of the samples, due to the shortening and separation of the fibers during the homogenization treatment, which reduces the projected area of the analyzed particles [18]. In addition, an increase in the fibrillation is observed in all sources with an increase in the slope of Figure 4 with the homogenization. To quantify this parameter, the values of BI have been collected in Table 5 in which the increase in the slope is translated into an increase in the value of this index. The smaller size of leek and artichoke results in more crossovers between fibers with a higher BI than eucalyptus and artichoke. Finally, by analyzing the BI of the different samples, it is possible to observe the differences between the homogenization passes. As we indicated with the micrograph characterization, the BI results after six and nine HPH steps are very similar, not requiring more than six homogenization passes, as the properties are maintained from this point on.

## 4. Conclusions

The production of highly concentrated LCMNF suspensions from greengrocery wastes without the extraction of the cellulose fraction is feasible. The presence of more extractives and shorter fiber lengths allowed obtaining leek LCMNF suspensions with a concentration of 5–5.5% and lettuce LCMNF suspensions with a 3.5–4% concentration, values much higher than those achieved in conventional CNF suspensions from woody plants. The best results are obtained when these greengrocery wastes are pretreated with the milling of the samples and their subsequent drying. In this state, it is possible to store the pretreated material in powder form for a long time before the production of LCMNF suspensions, occupying little space. Then, the powder is blended with water at high shear, and the suspension is homogenized up to six passes. However, the artichoke waste or the bleached eucalyptus (considered as the reference pulp, as it is traditionally used in the production of CNFs), with longer fibers and lower extractive content, do not allow for obtaining highly concentrated LCMNF suspensions. Besides the promising results, further studies are necessary to assess the effect of cellulose branching on different applications, such as in the preparation of emulsions in which immiscible phases are mixed and stabilized.

## Figures and Tables

**Table 1:** Chemical composition of the lettuce, leek, and artichoke waste and eucalyptus pulp.

	Lettuce	Leek	Artichoke	Eucalyptus Pulp
Dry content (%)	5.3 ± 0.3	11.2 ± 1.5	28.7 ± 2.8	Dried
Carboxyl groups (mmol/dry g)	2.51 ± 0.23	0.76 ± 0.07	1.01 ± 0.21	0.05 ± 0.01
**Chemical composition on a dry basis**				
Ash (%)	27.1 ± 0.4	10.8 ± 0.1	8.2 ± 0.2	<1%
Extractives (%)	14.4 ± 0.9	9.2 ± 0.7	1.8 ± 0.2	1.1 ± 0.3
Galacturonic acid (%)	8.3 ± 0.2	10.2 ± 0.2	15.4 ± 0.3	7.7 ± 0.7
Rhamnose (%)	1.4 ± 0.1	1.6 ± 0.1	1.3 ± 0.1	<0.3%
Cellulose (%)	17.7 ± 0.3	31.2 ± 0.6	28.2 ± 0.3	66.5 ± 0.2
Overlapping of xylose, mannose, and galactose (%)	5.0 ± 0.3	9.6 ± 0.2	10.9 ±0.1	13.6 ± 0.1
Arabinose (%)	0.8 ±0.1	2.0 ± 0.1	2.5 ± 0.1	<0.1%
Acid-soluble lignin (%)	20.0 ± 0.4	20.5 ± 0.7	15.9 ± 0.2	8.5 ± 0.4
Acid-insoluble lignin (%)	5.2 ± 0.4	4.9 ± 0.4	15.8 ± 0.4	1.3 ± 0.2

**Table 2:** Characterization of LCMNF suspensions pretreated with a blender (B) or dried, milled, and blender (DMB).

Pretreatment	Maximum Consistency after HPH	Transmittance 600 nm (0.1%)		Aspect Ratio	
		Before HPH	After HPH	Before HPH	After HPH
Lettuce					
Blender (B)	1.2–2%	24.5 ± 0.3	14.8 ± 0.2	49 ± 2	55 ± 2
Dry + Milled + Blender (DMB)	3.5–4%	16.5 ± 0.2	11.2 ± 0.2	36 ± 3	45 ± 2
Leek					
Blender (B)	1.3–1.5%	30.1 ± 0.2	17.5± 0.3	38 ± 4	43 ± 4
Dry + Milled + Blender (DMB)	5–5.5%	24.6 ± 0.2	21.1 ± 0.2	31 ± 3	38 ± 2
Artichoke					
Blender (B)	Not pass	49.4 ± 2.4	-	37 ± 3	-
Dry + Milled + Blender (DMB)	0.9–1%	32.6 ± 2.2	12.4 ± 0.7	33 ± 3	38 ± 3
Eucalyptus					
Blender (B)	0.4–0.5%	25.3 ± 1.2	23.3 ± 1.1	66 ± 2	77 ± 2
Dry + Milled + Blender (DMB)	0.4–0.5%	19.9 ± 1.2	19.4 ± 1.9	57 ± 3	64 ± 3

**Table 3:** Characterization of LCMNF suspensions pretreated with dried, milled, and blender (DMB) and different homogenization passes.

	Amount of Solids (%)	Transmittance 600 nm (0.1%)	Polymerization Degree (Number of Monomers)	Aspect Ratio	Cationic Demand (meq/g)
Lettuce					
DMB—0 passes	4.5%	16.5 ± 0.2	336 ± 22	36 ± 3	235 ± 30
DMB—1 pass	3.6%	11.2 ± 0.2	265 ±12	45 ± 2	-
DMB—3 pass	3.2%	5.1 ± 0.1	263 ± 3	51 ± 2	296 ± 16
DMB—6 pass	3.2%	7.4 ± 0.1	249 ± 16	54 ± 2	282 ± 6
DMB—9 pass	3.4%	8.2 ± 0.1	235 ± 4	55 ± 2	400 ± 30
Leek					
DMB—0 passes	7.5%	24.6 ± 0.3	293 ± 5	31 ± 3	49 ± 2
DMB—1 pass	5.3%	21.1 ± 0.3	-	38 ± 2	61 ± 4
DMB—3 pass	5.5%	18.7 ± 0.2	289 ± 2	40 ± 2	96 ± 4
DMB—6 pass	5.4%	19.6 ± 0.1	290 ± 10	41 ± 1	100 ± 3
DMB—9 pass	5.5%	20.0 ± 0.1	296 ± 6	42 ± 1	133 ± 11
Artichoke					
DMB—0 passes	4.5%	32.6 ± 2.2	747 ± 15	24 ± 4	96 ± 2
DMB—1 pass	0.93%	12.4 ± 0.7	589 ± 13	33 ± 3	121 ± 4
DMB—3 pass	0.94%	9.6 ± 0.6	576 ± 7	38 ± 3	132 ± 1
DMB—6 pass	0.94%	7.4 ± 0.3	570 ± 10	41 ± 3	143 ± 1
DMB—9 pass	0.95%	6.5 ± 0.1	569 ± 3	45 ± 2	149 ± 1
Eucalyptus					
DMB—0 passes	1.3%	19.9 ± 1.2	1044 ± 36	57 ± 2	196 ± 23
DMB—1 pass	0.42%	19.4 ± 1.9	863 ± 23	64 ± 2	238 ± 11
DMB—3 pass	0.39%	19.3 ± 1.4	839 ± 8	69 ± 2	249 ± 11
DMB—6 pass	0.38%	18.4 ± 0.5	833 ± 27	80 ± 3	262 ± 22
DMB—9 pass	0.35%	13.7 ± 1.1	842 ± 17	89 ± 3	305 ± 9

**Table 4:** OM images and diameter characterization of LCMNF suspensions pretreated with dried, milled, and blender (DMB) and different homogenization passes.

	DMB + 1 Pass HPH	DMB + 3 Pass HPH	DMB + 6 Pass HPH	DMB + 9 Pass HPH
Lettuce				
OM	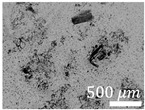	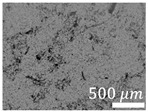	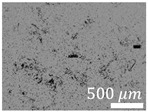	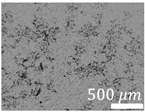
D10 (μm)	6.8	6.4	6.9	6.0
D50 (μm)	12.8	13.4	11.0	9.7
D90 (μm)	32	25.9	21.2	19.0
Standard deviation	34	8.9	6.9	6.4
Leek				
OM	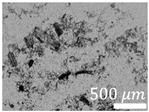	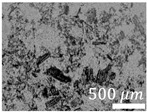	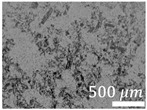	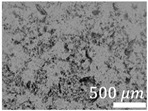
D10 (μm)	7.7	7.8	7.7	6.1
D50 (μm)	16.2	14.0	13.8	11.1
D90 (μm)	66	50	31	27.8
Standard deviation	27	28	13	11
Artichoke				
OM	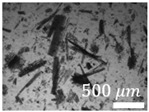	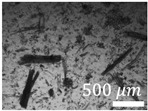	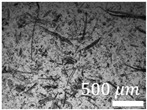	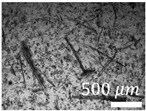
D10 (μm)	15.2	8.9	7.3	7.0
D50 (μm)	43	20.3	18.1	16.2
D90 (μm)	114	67	39	35
Standard deviation	47	28	12	13
Eucalyptus				
OM	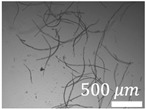	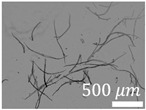	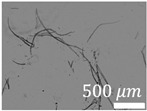	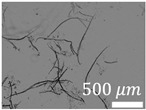
D10 (μm)	8.9	7.2	7.2	4.9
D50 (μm)	15.1	13.5	11.6	6.9
D90 (μm)	22.4	21.2	19.1	14.9
Standard deviation	6.2	5.3	5.1	4.1

**Table 5:** Branching index (BI) of LCMNFs produced with dried, milled, and blender (DMB) and different homogenization passes.

Branching Index (mm^−2^)	Eucalyptus (DMB-E)	Leek (DMB-P)	Lettuce (DMB-L)	Artichoke (DMB-A)
DMB + 0 passes HPH	699	3039	2844	828
DMB + 1 pass HPH	953	4567	3993	1481
DMB + 3 passes HPH	1153	4819	5046	1967
DMB + 6 passes HPH	2304	5405	5506	2447
DMB + 9 passes HPH	2291	5453	5590	2649

## Data Availability

Not applicable.
